# Relationship between Cumulative Temperature and Light Intensity and G93 Parameters of Isoprene Emission for the Tropical Tree *Ficus septica*

**DOI:** 10.3390/plants13020243

**Published:** 2024-01-15

**Authors:** Hirosuke Oku, Asif Iqbal, Shigeki Oogai, Masashi Inafuku, Ishmael Mutanda

**Affiliations:** 1 Tropical Biosphere Research Center, University of the Ryukyus, Okinawa 903-0213, Japan; okuhiros@comb.u-ryukyu.ac.jp (H.O.); k6730083@kadai.jp (S.O.); 2 The United Graduate School of Agricultural Sciences, Kagoshima University, Kagoshima 890-0065, Japan; asifiqbal1811@gmail.com; 3 Faculty of Agriculture, University of the Ryukyus, Okinawa 903-0213, Japan; h098648@agr.u-ryukyu.ac.jp

**Keywords:** isoprene emission, G93 formula, G93 algorithm, tropical tree, temperature response, light response, optimization

## Abstract

The most widely used isoprene emission algorithm, G93 formula, estimates instantaneous leaf-level isoprene emission using the basal emission factor and light and temperature dependency parameters. The G93 parameters have been suggested to show variation depending on past weather conditions, but no study has closely examined the relationship between past meteorological data and the algorithm parameters. Here, to examine the influence of the past weather on these parameters, we monitored weather conditions, G93 parameters, isoprene synthase transcripts and protein levels, and MEP pathway metabolites in the tropical tree *Ficus septica* for 12 days and analyzed their relationship with cumulative temperature and light intensity. Plants were illuminated with varying (ascending and descending) light regimes, and our previously developed Ping-Pong optimization method was used to parameterize G93. The cumulative temperature of the past 5 and 7 days positively correlated with *C_T_*_2_ and α, respectively, while the cumulative light intensity of the past 10 days showed the highest negative correlation with α. Concentrations of MEP pathway metabolites and *IspS* gene expression increased with increasing cumulative temperature. At best, the cumulative temperature of the past 2 days positively correlated with the MEP pathway metabolites and *IspS* gene expression, while these factors showed a biphasic positive and negative correlation with cumulative light intensity. Optimized G93 captured well the temperature and light dependency of isoprene emission at the beginning of the experiment; however, its performance significantly decreased for the latter stages of the experimental duration, especially for the descending phase. This was successfully improved through separate optimization of the ascending and descending phases, emphasizing the importance of the optimization of formula parameters and model improvement. These results have important implications for the improvement of isoprene emission algorithms, particularly under the predicted increase in future global temperatures.

## 1. Introduction

Due to their importance in regional and global atmospheric chemistry, biogenic volatile organic compounds (BVOCs) from terrestrial vegetation require accurate estimations to predict their current and future impacts on the warming climate. Constituting roughly 50% of BVOCs, isoprene (2-methyl-1,3-butadiene) is the most abundant BVOC emitted in copious amounts by terrestrial plants, imposing a huge impact on tropospheric chemistry [1,2]. In plants, isoprene has biological roles in the thermal protection of the photosynthetic apparatus and as a signaling molecule in gene and protein expression pathways related to stress response and plant growth [3,4,5,6,7,8,9,10,11]. In the atmosphere, however, isoprene is very reactive and readily undergoes radical-mediated photochemical reactions with the hydroxyl radical (OH) in the presence of nitrogenous oxides to generate toxic ground-level ozone and reactive intermediate species with far-reaching ramifications on tropospheric oxidative capacity [12,13,14]. In addition, isoprene (photo-) oxidation is the most significant contributor to the formation of secondary organic aerosols (SOAs), which act as cloud condensation nuclei, alter the global SOA budget, and cause severe air pollution and adverse health outcomes [14,15,16,17,18]. 

In plant leaves, isoprene is synthesized from dimethylallyl diphosphate (DMADP) supplied by the plastidial 2-C-methyl-D-erythritol 4-phosphate (MEP) pathway [19,20,21] using newly fixed carbon and by reducing power supplied from photosynthesis [22,23]. Though a number of factors are known that control isoprene emissions from plants, the most important environmental factors that drive isoprene emission are temperature, light intensity [24,25,26], and intercellular CO_2_ [27,28]. Most existing models consider the impact of these driving variables on isoprene emission [29,30,31,32]. The Guenther 1993 (G93) algorithm [33] simulates instantaneous leaf-level isoprene emission as a function of temperature and light drivers and is the most extensively used algorithm for the estimation of isoprene emissions globally. Despite the successive improvements in the model over the years, the temperature and light dependence formulas of G93 (C_T_ and C_L_, respectively) have remained essentially unchanged, except for the addition of past weather correction terms in the latest versions [1,2,34,35]. These formulas have been applied for estimating emissions from vegetation from all regions: low, mid, and northern high latitudes [36,37,38,39,40]. However, there have been cases where the algorithm under- or overestimated emission or completely deviated from observations, especially for tropical plants exposed to higher temperatures or light [41,42,43,44]. 

In an effort to improve the algorithm, we previously developed a method (named “Ping-Pong” method) to optimize the temperature and light dependency parameters of G93, which improved its performance for tropical trees [44]. The optimized G93 (O-G93) has since been applied to several other tropical species under varying temperature and light regimes [45] and for assessing the effect of growth conditions on sunlit and shaded leaves [46] with satisfactory robustness. Correlation analysis of our previous data set also revealed that past weather conditions have an influence on the parameters of G93, most probably through their effect on MEP pathway metabolites [46]. The average air temperature of the past 48 h, average photosynthetic photon flux density (PPFD), and basal emission factors correlated with C_T2_ and α parameters. 

Past weather history was previously demonstrated to impact not just isoprene emission capacity but also the temperature and light response of emissions. Monson et al. [25] grew velvet beans under warm (34 °C) or cold (26 °C) day temperatures and observed that plants grown under warm environments exhibited a higher optimum temperature (45 °C) for isoprene emission than those grown under cooler conditions (40 °C). Similar observations were reported for several other plants [47,48,49,50]. A critical question to consider is the number of days that plants must be exposed to certain whether conditions to impact isoprene emission, and weather cumulative temperature/PPFD of the previous days is vital in determining temperature/light response and basal emission capacities. Pétron et al. [51] demonstrated that isoprene emission capacity in *Quercus macrocarpa* doubled when growth temperatures of the preceding 3–6 weeks increased from 25 to 30 °C, while a decrease in growth temperature from 25 °C to 20 °C for 10 days caused a 25–50% decrease in isoprene emissions. Analysis of data from field studies with oaks also came to similar findings that the past weather history of the past 2 days is important for isoprene emission [49], which follows that the weather history needs to be captured in emission algorithms to improve their accuracy.

Despite the fact that temperature and PPFD of the previous 24 h (1 day) and 240 h (10 days) have already been incorporated in CT and CL of complex Guenther models such as MEGAN [1,2], as well as temperature of the past 15 days in G-99 [35], no study has closely examined the relationships between weather history and the parameters of the G93 formula that forms the basis of the MEGAN algorithms. We herein tracked the changes in G93 parameters of the same leaves of *Ficus septica* grown under natural and laboratory conditions for 12 days and examined the correlation between cumulative temperature and light intensity, O-G93 parameters, the level of MEP pathway metabolites, isoprene synthase protein, and gene expression levels. We report that although our O-G93 demonstrated improved estimations of emissions compared with the default G93 and the light and temperature algorithms of MEGAN, it also displayed limited performance with an increase in cumulative temperature of previous days at the latter stages of the experiment. The deviation was most pronounced in the latter stages of the experimental duration due to a shift in isoprene emission peak likely caused by cumulative temperature of the previous days. Thus, we herein report an improved approach that involves separate optimization of ascending and descending phases (simulating typical diurnal emission patterns) that improved estimations by O-G93 to capture previous weather history. Our result has important implications for the improvement of isoprene emission algorithms especially under the predicted warmer future climate.

## 2. Results

**Figure 1:**
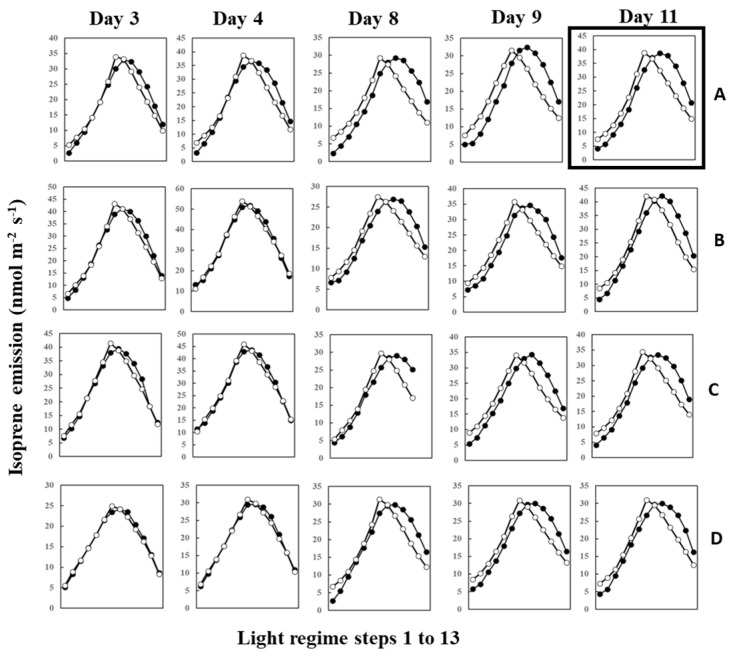
Observed isoprene emission (⬤) and prediction by optimized G93 (○) of leaves (**A**–**D**) during the experimental duration. Measurements were conducted for days 3, 4, 8, 9, 10, and 11, but the illustration for day 10 was omitted in the figure because it showed an almost similar profile to day 9 or day 11. Data of leaf A on day 11 (boxed) were further used in the next section to compare the efficacy of MEGAN and G93 emission models.

During measurements, leaves were irradiated with 13 steps of an up and down lighting program, as illustrated in Supplementary Figure S1A, giving a light intensity range of 180 to 1300 μmol m^−2^ s^−1^. Leaf temperature was allowed to vary naturally with light intensity to imitate a real field scenario; thus, it also defined an up and down curve (Supplementary Figure S1B). Figure 1 shows the light- and temperature-dependent isoprene emission from four leaves (leaves A–D) starting from 12 June (Day 3) to 20 June (Day 11) of 2020. Isoprene emission peaked in step 8 or 7 for all four leaves on 12 June (Day 3) and 13 June (Day 4). We used O-G93 to estimate emissions on all the days, and Figure 1 shows that O-G93 largely captured well the light and temperature dependence of isoprene emission on days 3 and 4 for all leaves. However, from day 8 onwards, the emission peak shifted to step 9, and predictions by O-G93 deviated from the observed emission rate from this day until day 11 in all four leaves, as shown in Figure 1. Compared with the ascending phase, the deviation was more pronounced for the descending phase in all cases. 

**Figure 2:**
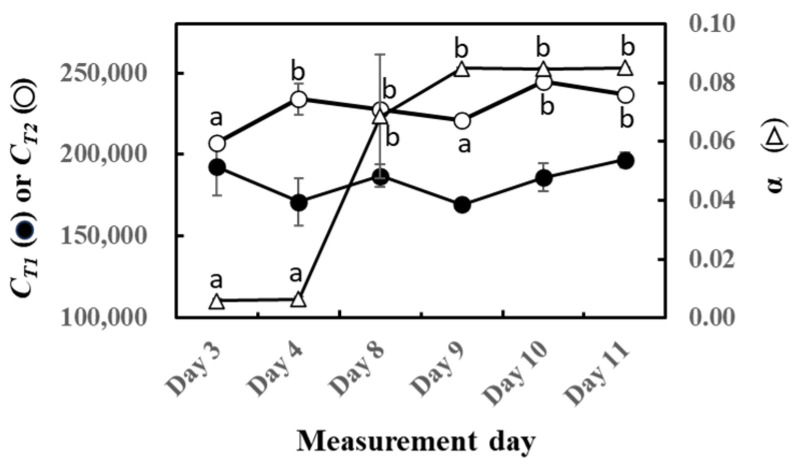
Changes in *C_T_*_1_ (○), *C_T_*_2_ (⬤), and α (△) of optimized G93 during the experimental duration. The results of the post hoc analyses were shown only when the initial ANOVA test yielded statistically significant results (*p* < 0.05). Data are mean ± SE of 4 analyses. Data sharing different letters are significantly different at *p* < 0.05.

The parameters of O-G93 (C_T1_, C_T2_, and α) were calculated for the experimental duration, and their performance on capturing the light and temperature dependence is shown in Figure 2. No statistically significant change was noted with *C_T_*_1_, in agreement with our previous study on sun and shade leaves of four tropical trees [46]. In contrast, *C_T_*_2_ significantly increased with the laps of the experiment. Most distinctive changes were noted with the parameter α that increased dramatically towards the end of this experiment.

To gain more insights into the relationship between past weather history and O-G93 parameters, correlation analysis was performed (Table 1). Again, no statistical significance was found between *C_T_*_1_ and average temperature of the past days (up to 10 days). However, *C_T_*_2_ positively correlated with the average temperature of the past 2 to 9 days, with the highest correlation (0.65) for the average temperature of the past 5 days. The alpha of O-G93 also showed highly positive correlations with the average temperature of the past days, with the highest correlation coefficient (0.90) for the average temperature of the past 7 days.

**Figure 3:**
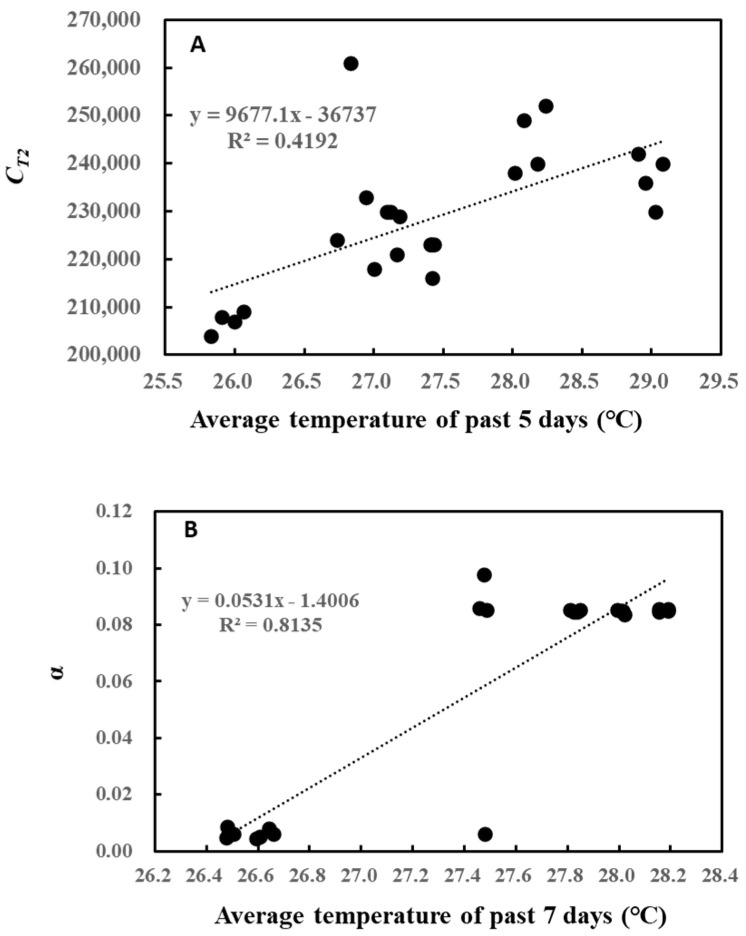
Correlation between *C_T_*_2_ and average temperature of the past 5 days (**A**) and between α and average temperature of the past 7 days (**B**).

A closer look at these relationships shows that *C_T_*_2_ linearly increased with the average temperature of the past 5 days (Figure 3A), while α also followed a similar increase with the average temperature of the past 7 days (Figure 3B). However, the increase for α seemed to be somewhat stepwise, with the threshold air temperature around 27 °C.

**Figure 4:**
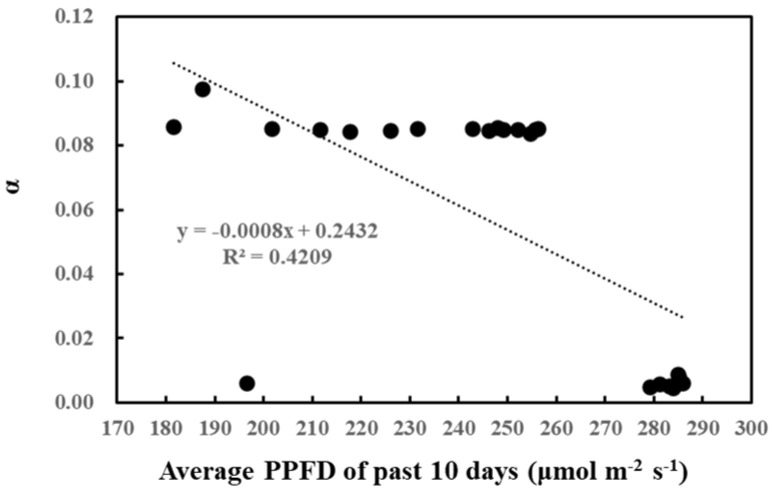
Correlation between α and average PPFD of the past 10 days.

Likewise, the correlation between cumulative past light intensity and O-G93 parameters was studied (Table 2). The average of past light intensity showed no correlation with *C_T_*_1_, while *C_T_*_2_ displayed a positive correlation with the average light intensity of the past 5 days. In contrast, α negatively correlated with the average PPFD of the past 8, 9, and 10 days. This negative correlation also seemed to be stepwise rather than linear, with the threshold of the average daily light intensity around 260 µmol/m^2^/s (Figure 4). A daily average light intensity of less than 260 µmol/m^2^/s for 10 days boosted up α to around 0.08.

**Figure 5:**
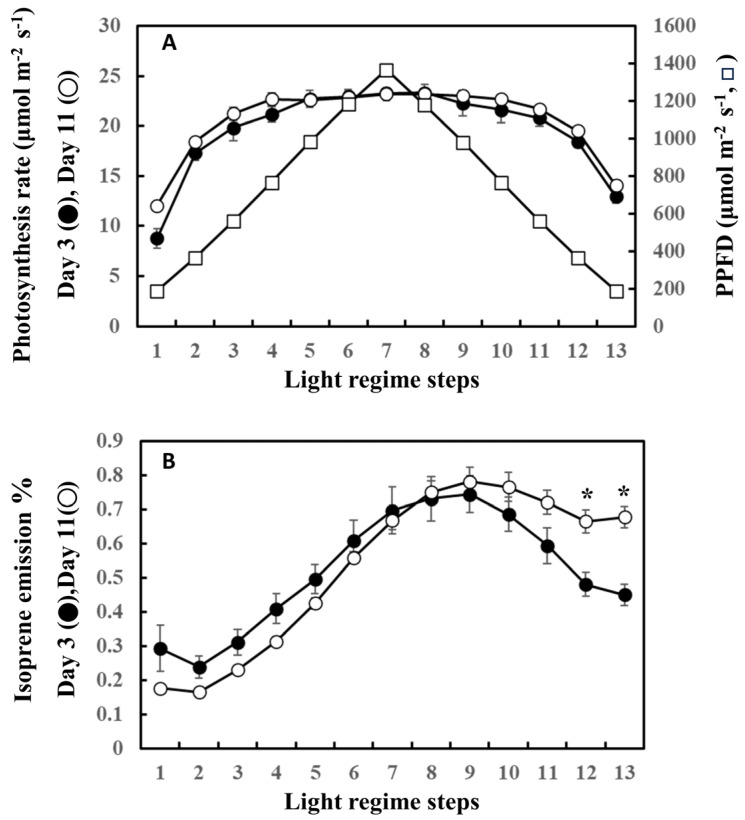
Comparison of photosynthesis rate (**A**) and the ratio of carbon emitted as isoprene to carbon fixed by photosynthesis (**B**) between day 3 and day 11. For clarity, comparison between day 3 (beginning of the experiment) and day 11 (end of the experiment) was performed. Data are mean ± SE of 4 analyses. Asterisks denote statistically significant differences between day 3 and day 11 by the Tukey test.

The precursor of isoprene biosynthesis, DMADP, is supplied by photosynthesis through the MEP pathway. Figure 5 shows the photosynthesis rate and the percentage of photosynthetically fixed carbon that is emitted as isoprene on the first (day 3) and last (day 11) days of the experiment. For clarity, data from day 3 and day 11 only are shown. No significant differences were noted in the rate of photosynthesis between day 3 and day 11 throughout the steps of ascending and descending phases (Figure 5A). However, the percentage of carbon emitted as isoprene on day 3 was higher than that on day 11 during the ascending phase, while it was surpassed by day 11 after step 8. The percentage of carbon lost as isoprene on day 11 was significantly higher than that on day 3 in steps 12 and 13, suggesting a higher input of fixed carbon for the descending phase on day 11.

**Figure 6:**
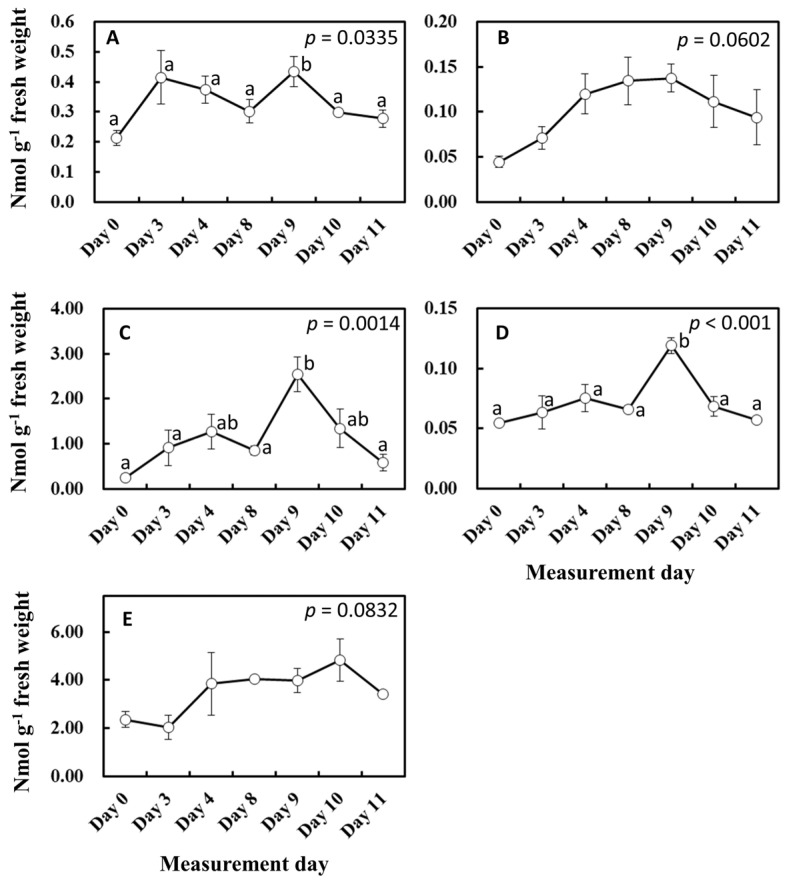
Changes in MEP pathway metabolites. Result of day 0 was included in these data. The results of the first ANOVA were shown in the figure, and the post hoc analyses were presented only when the initial ANOVA test yielded statistically significant results (*p* < 0.05). Data are mean ± SE of 4 analyses. Data with different letters are significantly different at *p* < 0.05. (**A**) DXP = 1-deoxy-D-xylulose-5-phosphate, (**B**) MEP = methylerythritol 4-phosphate, (**C**) MEcDP = 2-C-methyl-D-erythritol-2,4-cyclodiphosphate, (**D**) HMBDP = 4-hydroxy-3-methylbut-2-enyl-diphosphate, and (**E**) DMADP = dimethylallyl diphosphate.

**Figure 7:**
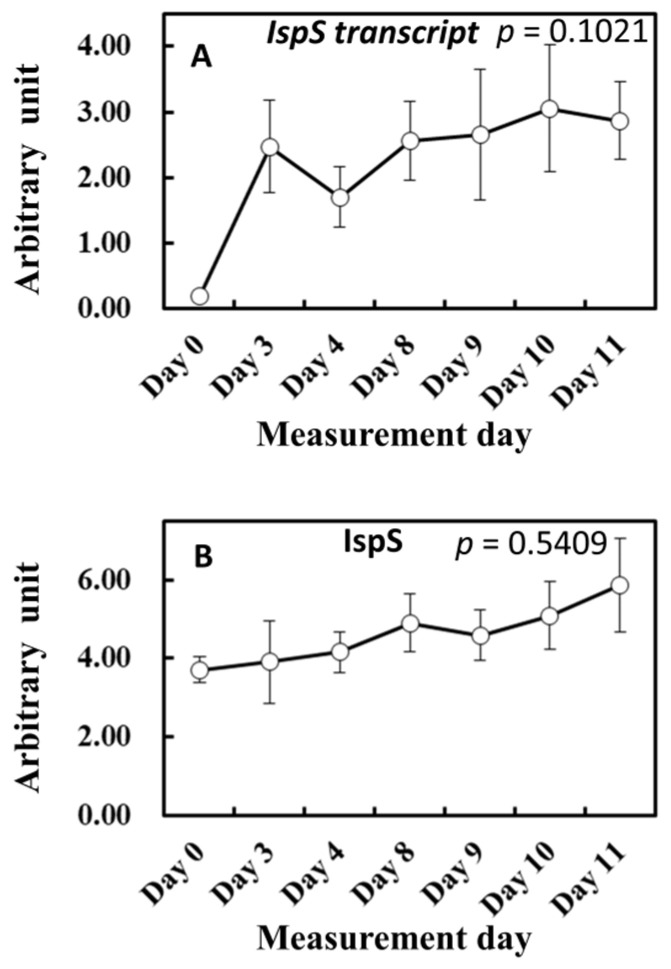
Changes in *IspS* transcript (**A**) and IspS synthase level (**B**). The results of the first ANOVA are shown in the figure. Data are mean ± SE of 4 analyses.

To gain more insights into the influence of light and temperature history on substrate and enzyme availability for isoprene biosynthesis, MEP pathway metabolites, isoprene synthase (*IspS*) gene expression, and IspS protein levels were measured from the leaf samples growing in the vicinity of leaves A to D throughout the experiment (Figure 6 and Figure 7). MEP and DMADP pools increased after day 3 compared with day 0 (*p* = 0.060 and *p* = 0.083, respectively). 1-Deoxy-D-xylulose-5-phosphate (DXP), 2-C-methyl-D-erythritol-2,4-cyclodiphosphate (MEcDP), and 4-hydroxy-3-methylbut-2-enyl-diphosphate (HMBDP) significantly increased from day 0, peaking on day 9, and thereafter decreased towards day 11 (Figure 6). Compared with day 0, the gene expression of *IspS* increased sharply on day 3 but remained roughly at the same level towards day 11 (Figure 7A). IspS levels appeared to increase gradually towards day 11, but the changes were not statistically significant during the entire period of the experiment (Figure 7B).

To highlight the links between past weather conditions (cumulative temperature and light intensity) and MEP metabolites, *IspS* gene expression, and IspS protein levels, correlations between the conditions and these parameters were studied. Table 3 lists the correlation coefficients between past average temperature and MEP metabolites, *IspS* gene expression, and IspS synthase levels. The average temperature of the preceding 1 to 3 days positively correlated with DXP, MEP, MEcDP, and DMADP metabolites. Among the assayed metabolites, only HMBDP showed no statistically significant correlation with cumulative average temperature. *IspS* gene expression was positively correlated with the average temperature of the preceding 1 to 3 days. IspS protein level is likely to increase with the average temperature of the past 5 days (*p* = 0.054) even though no statistical significance was reached probably due to large fluctuations in the data (Figure 7B). In general, all correlation coefficients decreased with the increasing time period of the past days.

Likewise, Table 4 lists the correlation coefficients between past average PPFD and concentration of MEP pathway metabolites, *IspS* gene expression, and IspS protein levels. The correlation of cumulative light intensity with these parameters was biphasic: the average of the preceding 1 or 2 days positively correlated with MEP pathway metabolites and *IspS* expression, while the average of the past 6 or more days showed negative correlation with the concentration of metabolites and *IspS* gene expression. IspS protein levels showed no significant correlation with the average PPFD of the previous days.

Our current study focused on the effect of past weather history on leaf-level isoprene emissions. In this regard, the later versions of the Guenther emission algorithms incorporated past weather effects into the temperature and light intensity response formulas in the MEGAN model [1,2]. The MEGAN model uses the input of the past average 24 h (1 day) and 240 h (10 days) for both temperature response (*γT*) and light response (*γP*) simulation formulas (Supplementary Document S1). Thus, isoprene emission is normalized by *γT* × *γP* in terms of temperature and light response. To enable comparisons among MEGAN, G93, and optimized G93 (O-G93), we herein implemented the MEGAN model with the influence of the past weather, but without most of the driving environmental variables (soil moisture, leaf age, and CO_2_). We then compared the efficacy of this temperature and light response of MEGAN algorithms with O-G93 and default G93 (*C_T_* × *C_L_* in this case) using isoprene emission of leaf A on day 11 as a typical case (please refer to Figure 1). 

**Figure 8:**
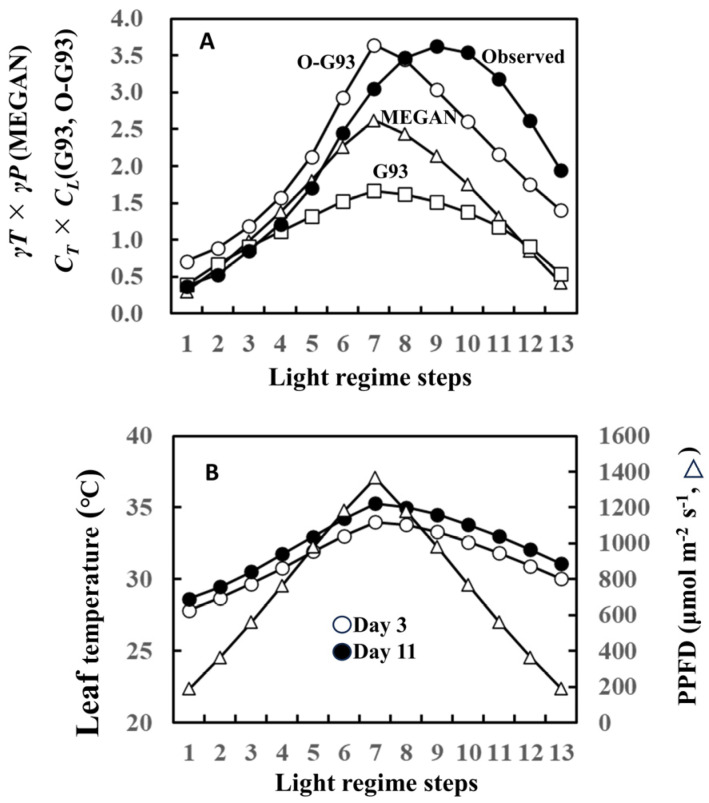
Comparison of temperature and light response factors between optimized G93, G93, and MEGAN formula (**A**) and comparison of leaf temperature and PPFD between day 3 and 11 (**B**) using data of leaf A on day 11 as a typical case of latter-stage isoprene emission and prediction. MEGAN describes the temperature- and light-dependent leaf-level isoprene emission as *γP* and *γT* (Supplementary Document S1) corresponding to *C_T_* and *C_L_* of G93. Temperature and light responses of observed emission were estimated based on basal emission rate determined by optimized G93 (observed emission rate was divided by basal emission rate to give the estimation of *C_T_* × *C_L_*).

The temperature and light response of the MEGAN algorithm failed to capture isoprene emission flux of this day, as well as our O-G93 and default G93 (Figure 8A). The largest deviations of the predicted curves from the observed response curve were more evident for the descending phase in all cases, with a clear shift of the maximal emission step. As described in the result of Figure 1, the maximal emission of leaf A on day 3 was observed in step 7 or 8, while the maximum emission shifted to step 9 on day 11. This could imply that there was a change in the leaf temperature response profile between day 3 and day 11. We therefore compared leaf temperature with varying PPFD steps for the 2 days, and the data show no difference in the shapes of the symmetric curves for the ascending and descending phases on both days (Figure 8B). The leaf temperature on day 11 was 0.8 to 1.3 °C higher than on day 3 throughout the steps (Figure 8B), and furthermore, the temperature difference peaked in step 7 with a roughly similar curve shape, as shown for O-G93 in Figure 8A. Changes in leaf temperature sensitivity, therefore, could not explain the deviation of prediction from the observed temperature and light response of flux on day 11. 

**Figure 9:**
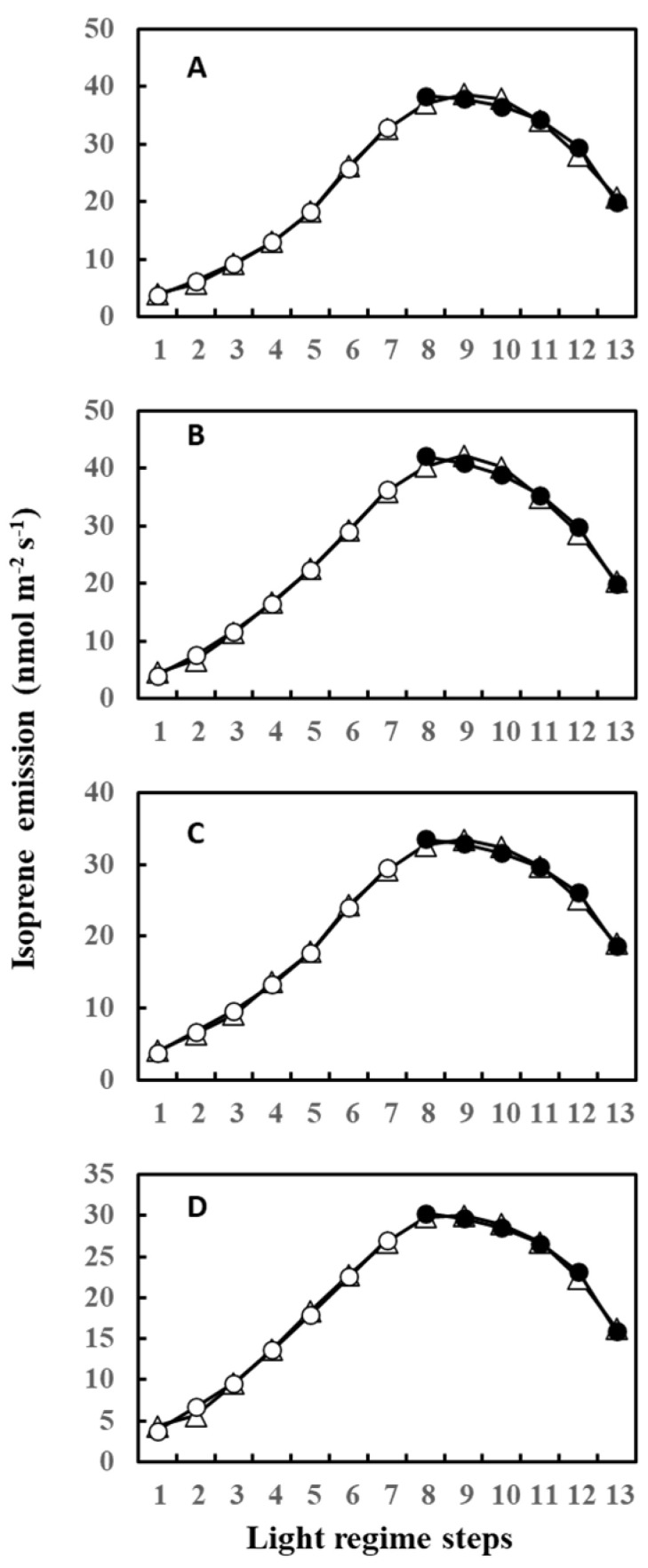
Observed isoprene emission (△) and prediction of ascending phase (○) and descending phase (⬤) emission by an individually optimized G93 formula for leaves on day 11 presented in Figure 1. (**A**–**D**) denote leaves (**A**–**D**) shown in Figure 1.

All three models—MEGAN, default G93, and O-G93—applied the same parameters for the temperature and light response of both the ascending and descending isoprene emission phases, assuming that the temperature and light response across the maximal light intensity remained the same. This seems to be applicable for the tropical plant *F. septica* under relatively moderate weather conditions, such as occasional rains, but not in the case of continuous hot weather as exemplified in this study. We thus used the Ping-Pong method to separately optimize the parameters of G93 for the ascending and descending phases to better capture the isoprene emission flux on day 11 (Figure 9). A separate optimization of the ascending and descending phases successfully captured the observed emission rate on day 11 for all leaves.

Table 5 summarizes the optimized parameters of G93 with whole data, including both the ascending and descending phase (termed “whole” here) and those where the ascending and descending phases were optimized separately. The *C_T_*_1_, *C_T_*_2,_ and basal emission rate (BER) of the ascending phase were comparable to those of “whole”, while the α of the ascending phase was significantly lower than that of “whole”. In contrast, all parameters of the descending phase differed from “whole”. The *C_T_*_1_ and α of the descending phase were lower by 4 and 22 times, respectively, while *C_T_*_2_ and BER were higher than those of “whole”. More importantly, the *C_T_*_1_ and *C_T_*_2_ of the descending phase differed from those of the ascending phase except for α, suggesting that individual parameterization for the ascending and descending phases is needed in the case of hot weather history. Applying the same approach to the data of day 3 revealed no differences in the parameters between the ascending and descending phases (Supplementary Table S1).

## 3. Discussion

The relationship between weather history and isoprene emission behavior was studied in the broad-leaf tropical tree *F. septica*. The effect of past weather on isoprene emission response has been known for some time [25,49,50], and past meteorological data have been incorporated into emission algorithms as variables to improve model performance [1,2]. However, no study has closely examined the relationship between past weather and the parameters of these algorithms. Our previous study found that the O-G93 parameters of four tropical trees correlated with cumulative temperature or light intensity [46]. The current study refined our previous approach and further examined more closely the relationship between temperature or light history and parameters of G93 to explore the environmental control of isoprene emission. To gain more insights into how past weather conditions influence the isoprene emission behavior of *F. septica*, isoprene emissions of the same leaves were monitored for 12 days starting from cooler weather conditions to allow a progressive increase in cumulative temperature and PPFD towards day 11. 

Four leaves (A to D) displayed very similar changes in predicted and observed emission profiles throughout the study period. The O-G93 formula captured well the actual emission rate on days 3 and 4 but deviated from observed emission fluxes after day 8 (Figure 1). The deviation was most pronounced in the descending phase of light response for all four leaves (Figure 1). This deviation was reflected in the changes in optimized parameters for G93, as illustrated in Figure 2. Most distinctive changes were noted with α. The parameter α often increased to around 0.08 in our previous study with some tropical trees, similar to observations in this study. The iterative method named “Ping-Pong” optimizes the parameters *C_T_*_1_, *C_T_*_2_, and α to minimize the errors between observed and predicted emission rate [44]; thus, large increases in α after day 8 can be explained by the maximization of efficacy of *C_L_*. *C_L_* is a function of α and reached almost a plateau level at around α = 0.03 (Supplementary Figure S2). Changes in α beyond 0.03, therefore, have little or negligible practical impact on the final *C_L_* value, with changes in *C_L_* probably less than 0.005 beyond α = 0.03. The high values of α greater than 0.03 are mathematically accurate and correct but considered practically equivalent in their impact on the final *C_L_* value, and appeared to be linked with the poor performance of O-G93 to predict emission rate from day 8.

The poor performance of all emission formulas, O-G93, default G93, and MEGAN after day 8 is associated with the observed shift in maximal emission steps that caused an asymmetry of emission profile across the maximal light intensity or temperature, as exemplified in Figure 8A. The maximal light intensity and leaf temperature under our measurement conditions was in step 7 for all experiments (Figure 8B). At the beginning of the experiment (days 3 and 4), the maximal emission rate coincided with PPFD and leaf temperature peak, as expected, in step 7 or 8. The gap between maximal irradiance (step 7) and emission peak (step 8) could be due to a time lag between the onset of emission and adjustments in photosynthesis rate at a given light regimen to supply substrate and energetic cofactors [52,53]. O-G93 captured well the observed emission rates under these conditions. However, the peak emission for periods after day 8 shifted to step 9 and remained at almost the same level in step 10 for all leaves under exactly the same experimental conditions. Due to this shift, all emission formulas based on the assumption that isoprene emissions for both ascending and descending phases are simply dependent on light intensity and temperature failed to capture the actual emission rate, as shown in Figure 8A. Conversely, the parameters may depict variation between ascending and descending phases and need revision for separate optimization after day 8.

The individual optimization of ascending (steps 1 to 7) and descending phases (steps 8 to 13) successfully captured the observed emission rate, as exemplified for day 11 (Figure 9). The parameters of the ascending phase, except for α, were comparable to those of “whole” that parameterized ascending and descending phases together (steps 1 to 13) (Table 5). On the other hand, all parameters of the descending phase differed from “whole”: the *C_T_*_1_ and α of the descending phase were lower, while the *C_T_*_2_ and BER were higher than those of whole. More importantly, the parameters of the descending phase differed from those of the ascending phase except for the parameter α, suggesting that parameterization should be performed separately for the ascending and descending phases for the leaves of *F. septica* during periods of high cumulative temperature or PPFD. 

Cumulative temperature modulated the *C_T_*_2_ of O-G93 (Figure 2), as was the case with our previous observation in that *C_T_*_2_ positively correlated with the average temperature of the past 2 to 5 days [46]. In that study, the increase in *C_T_*_2_ with cumulative past temperature was explained to show a higher temperature dependency of isoprene emission. This explanation appears to hold true in the current study as well. An increase in the average temperature of the past 5 and 7 days had the highest correlation with the *C_T_*_2_ and α of O-G93, respectively (Table 1 and Figure 3). Thus, the increase in *C_T_*_2_ may reflect the high temperature response to cope with the coming hot weather. The cumulative temperature of the past 7 days also showed a statistically significant correlation with α. However, α appeared to change stepwise from around 0.005 to 0.085 with the cumulative temperature of the past 7 days (Figure 3B). This stepwise jump in α rather coincided with the shift in the emission peak as discussed above and can be taken as a hallmark for the limited performance of O-G93. Thus, Figure 3B may suggest an occurrence of a threshold cumulative past temperature for a peak shift of around 27.2 °C for *F. septica*. The threshold temperature may show variation within species because our previous study found an average of α = 0.0056 for four tropical trees (*Syzygium cumini*, *S. samarangense*, *Bauhinia variegata*, and *Mangifera indica*) despite an average temperature of 28.5 °C for the previous 7 days [46]. *F. septica* may be more susceptible to hot weather and alter its isoprene emission behavior compared with other species. This needs further testing with an increased number of tropical trees.

**Figure 10:**
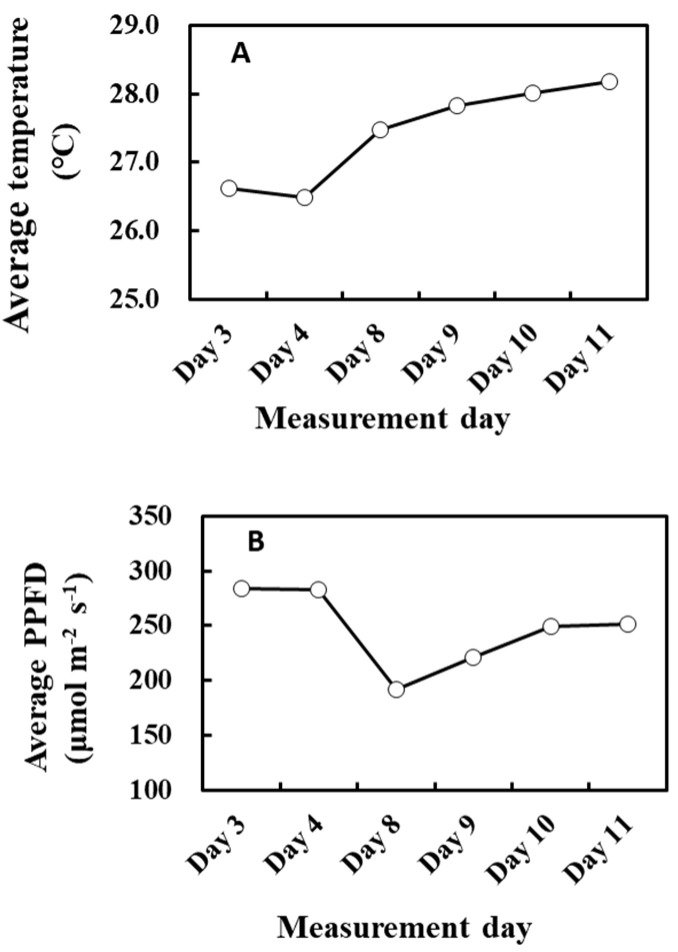
Average air temperature of the past 7 days (**A**) and average PPFD of the past 10 days (**B**) during the experiment.

There was a negative correlation between the cumulative light intensity and α of O-G93 (Table 2 and Figure 4). The correlation profile resembles that of α and cumulative temperature (Figure 3B) in that the change in α was stepwise, but different in that the correlation between cumulative PPFD and α was negative. Moreover, the correlation was statistically significant for more extended past periods (past 10 or possibly more days), which could reveal a considerable difference between the effects of past cumulative PPFD and temperature on α. This seemingly opposing relationship between past meteorological variables and G93 parameters is somewhat puzzling, so to tease the effects apart, we plotted the average past temperature (7 days) and average PPFD (10 days) through the measurement days (Figure 10). It is evident from the profiles that the average temperature of the past days increased towards day 11 (Figure 10A), while the average PPFD of the past 10 days decreased towards day 11 (Figure 10B), and that both changes were more drastic on day 8. Cumulative temperature had a higher positive correlation (Table 1) compared with cumulative PPFD (Table 2). It is thus plausible that these positive and negative effects under given environmental conditions could offset and balance each other, and the net effects are integrated into α. 

Isoprene is biosynthesized by IspS from DMADP supplied by the MEP pathway using newly fixed carbon and by reducing power from photosynthesis [22,23]. Consequently, the concentrations of metabolites and IspS mRNA, protein, and activity levels are factors that control isoprene emission rates, but their effect on isoprene emission at intermediate timescales is complex and does not always show direct correlations [50]. To test whether the substrate and reducing power supply or IspS levels could explain the shift in isoprene emission as observed between day 3 and days 8–11 (Figure 1 and Figure 5), we tracked photosynthesis rate, MEP metabolites, and *IspS* gene and protein levels. The emission measurements in the leaf chamber were conducted for short durations (about 1 h) under the same conditions; thus, IspS levels can be assumed to be comparable throughout the measurement procedure since IspS does not change instantaneously with heat/light stress over such a short timescale [54]. Photosynthesis rates were similar between days 3 and 11 (Figure 5A); however, the percentage of carbon emitted as isoprene exhibited differences between the days (Figure 5B). The isoprene emission percentage during the ascending phase on day 11 was consistently lower than that on day 3, while the percentage during the descending phase was significantly higher than that on day 3, suggesting a higher substrate concentration for the descending phase on day 11 even under the same light regimen. This view was supported by increased concentrations of MEP pathway metabolites such as DXP, MEP, and DMADP after day 8 (Figure 6). Concentrations of MEP metabolites, *IspS* gene expression, and IspS protein can be assumed to be regulated by cumulative weather conditions, although the relationship is complex and not straightforward [28]. As expected from the relationship between past weather history and isoprene emission, cumulative temperature up to the past 3 days positively correlated with the concentration of MEP pathway metabolites and *IspS* gene expression (Table 3). This suggests that *F. septica* leaves acquired high MEP pathway activity with an increase in cumulative temperature, probably to cope more rapidly with the anticipated hot weather. This may also explain the shift in emission peak and the redundancy of light or temperature dependence of isoprene emission for the descending light regime phase, as presented in Figure 1 and Figure 8A. 

**Figure 11:**
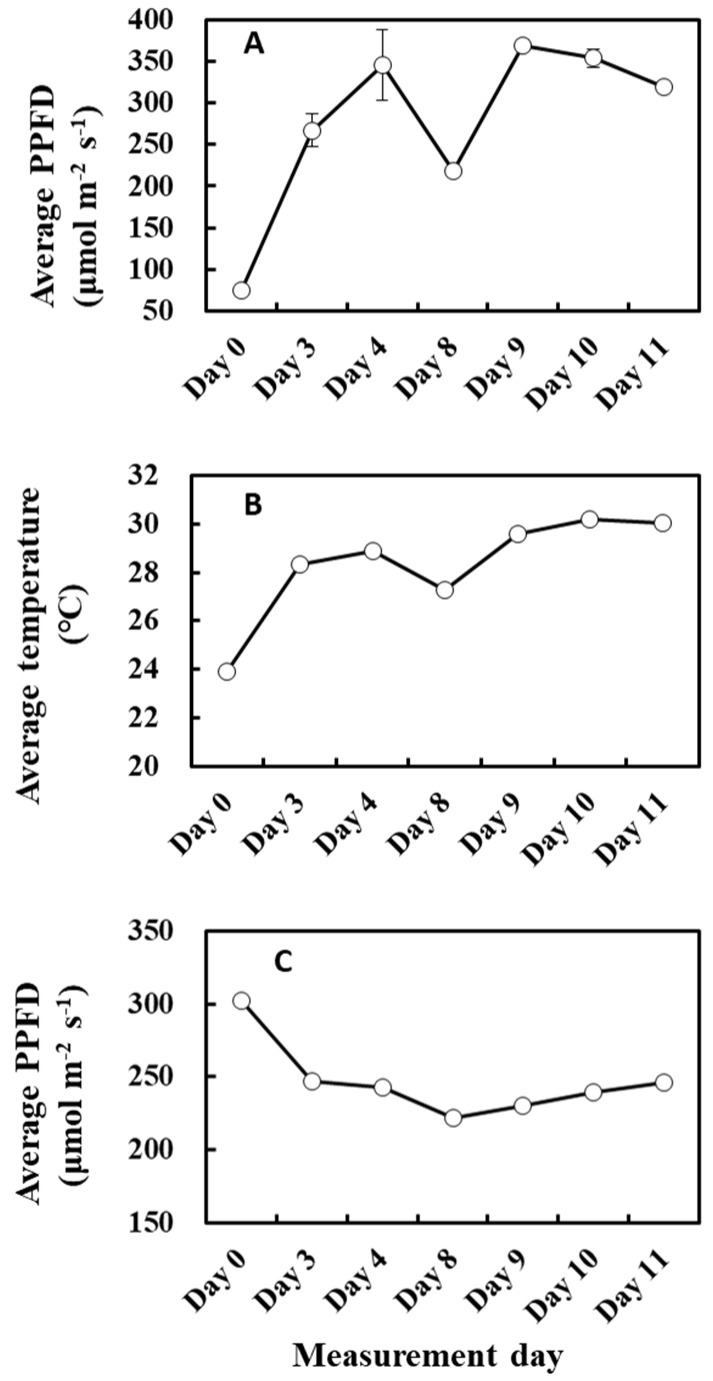
Average PPFD of the past 1 day (**A**), average temperature of the past 2 days (**B**), and average PPFD of the past 8 days (**C**) during the experiment. Data are mean ± SE of 4 analyses.

The relationship between cumulative PPFD and MEP pathway metabolites agrees in part with the above explanation because the light intensity of the previous day positively correlated with the concentration of metabolites and *IspS* gene expression (Table 4 and Figure 11A). However, the relationship between cumulative PPFD and MEP pathway metabolites or *IspS* gene expression was biphasic and showed a negative correlation for days longer than 6 (Table 4). This relationship is intriguing but somewhat puzzling. MEP pathway metabolites were positively correlated with temperature and increased with cumulative temperature up to 3 past days towards day 11 (Table 3 and Figure 11B), while cumulative PPFD of the past 8 days decreased (Figure 11C). Thus, it could be possible that MEP pathway flux was primarily driven by cumulative temperature rather than cumulative light intensity under the present circumstances, which resulted in a negative correlation between PPFD and MEP metabolite concentration. This is reminiscent of the opposite correlation case of temperature and light intensity on α discussed above (Figure 3 and Figure 4). Alternatively, it may suggest that an excess dose of light over a long duration could inhibit the activity of the MEP pathway due to increased photorespiration [55]. Overall, these data suggest teasing apart the effects of weather between 1 and 3 days and those for over 8 days.

**Figure 12:**
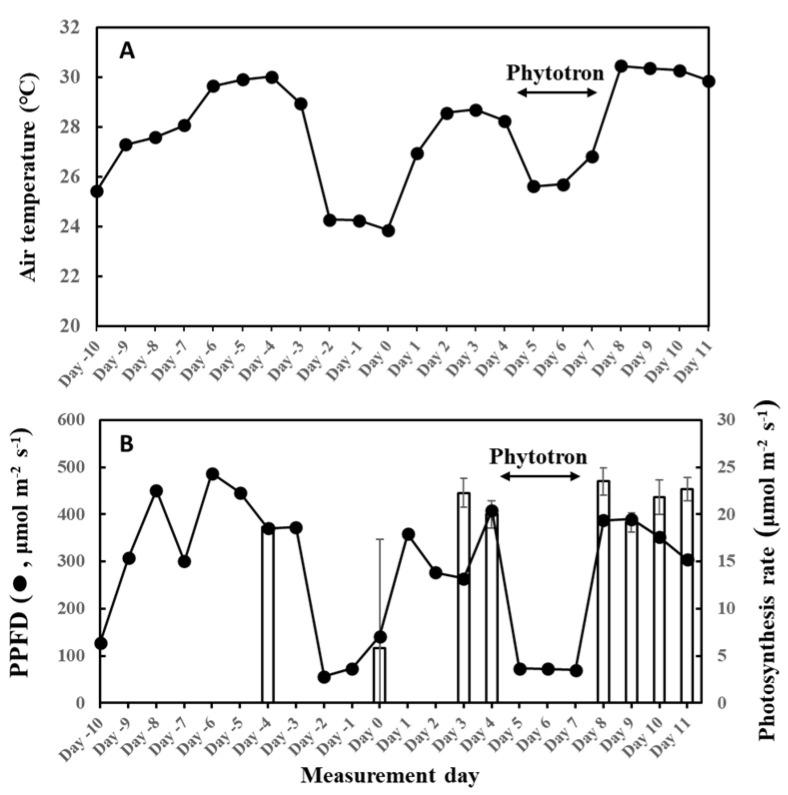
Air temperature (**A**) and light intensity and photosynthesis rates (**B**) during the experiment. Four, 2-year-old *F. septica* sapling clones growing in an open field or phytotron (days 5 to 7) were measured for 12 days. Isoprene emission measurements started on 9 June 2020 (Day 0), with an increase in cumulative temperature and light intensity, and ended on 20 June 2020 (Day 11). Photosynthesis rates (bar graph) were the average readings in steps 4 to 6 of the irradiance program with an average PPFD of 1000 µmol/m^2^/s, and repents mean ± SE of 4 analyses. The data for 5 June was the result of a preliminary measurement for reference (leaf C). Plants were grown in laboratory conditions from day 5 to 7 to manipulate growth conditions.

There was a complete stoppage of photosynthesis on three leaves on day 0 of this experiment, and there was no observed isoprene emission on those leaves—even the fourth leaf that had photosynthesis showed extremely low isoprene emission rates (Figure 12). Photosynthesis is the driving force of isoprene emission and appeared to be also dependent on weather history. We previously reported a threshold temperature of 12 °C to initiate isoprene emission from *F. septica* leaves [56], and showed that this control was at the IspS protein level [57,58]. The average temperature of the previous 2 days was 24.3 °C on day 0; thus, it was higher than the threshold temperature and therefore could not explain the cessation of isoprene emission. The interruption of photosynthesis explains the low MEP metabolites, including DMADP on the day (Figure 6). On day 0, the average PPFD of the previous 2 days was 65 μmol/m^2^/s, which could explain the cessation of photosynthesis if this is the threshold PPFD for photosynthesis in the species. However, on day 8 with high photosynthesis, the average PPFD of the previous 2 days was 71 μmol/m^2^/s, which is comparable to day 0. This observation warrants further examination to determine if there is a threshold PPFD between 65 and 70 μmol/m^2^/s for photosynthesis in *F. septica*. It is also likely that light intensity and temperature synergistically control photosynthesis at several levels.

## 4. Materials and Methods

### 4.1. Plant Materials

Four, 2-year-old *F. septica* sapling clones were used in this experiment. The plants, in 30 L plastic pots, were grown in an open field at the University of the Ryukyus in Okinawa, Japan (26°15′ N, 127°46′ E). Saplings were watered every 2 or 3 days to avoid drought stress during the experiment. Healthy mature leaves at the third or fourth nodes from the apex were used throughout. Isoprene emission was measured from the same individual leaf from each pot (total of 4 leaves) for 12 days between 9 June (termed day 0) and 20 June (termed day 11), 2020. For the analysis of MEP pathway metabolites and *IspS* transcript and protein levels, 1 neighbor leaf was harvested from the vicinity of the measurement leaf before emission measurements and immediately stored at −80 °C. 

### 4.2. Experimental Design

To assess the relationship between past weather conditions and G93 parameters, we measured isoprene emission starting from cooler temperatures to warmer days, with sufficient fluctuations in the weather conditions between the experimental period. Meteorological data were monitored way before isoprene emission measurements commenced, as shown in Figure 12. There were 2 rainy days from 7 to 8 June (days –2 and –1), and it was cloudy with occasional rains on 9 June (day 0), so emission measurements were commenced on 9 June (cooler, cloudy weather), as illustrated in Figure 12. The average temperature and PPFD for 9 June were 23.9 °C and 142 µmol/m^2^/s, respectively. Surprisingly, no photosynthesis was observed in 3 of the 4 leaves (leaves B, C, and D), and therefore, no isoprene emission was detected, while normal levels of photosynthesis and very low levels of isoprene emission were observed only on leaf A. For this reason, data from day 0 were not included for the optimization of G93 parameters. Isoprene emission was therefore measured on days 3 and 4 to ensure reliable measurements and parameterization of isoprene emission, and solid emission was observed on these days. The plants were then moved to a phytotron from days 5 to 7 (3 days) to manipulate temperature and light intensity because a fine weather was forecasted for the coming 7 days up to day 11. Figure 12 shows the changes in average temperature and light intensity throughout the entire experimental period. Measurements of isoprene emission and photosynthesis were conducted on days 3, 4, 8, 9, 10, and 11. For emission measurements, leaves were clamped in a leaf chamber, as described below, and illuminated for 13 steps of increasing and decreasing PPFD, as illustrated in Supplementary Figure S1A. Photosynthesis rates in Figure 12 were the averages of readings in steps 4 to 6 of the irradiance program (Supplementary Figure S1A) with an average light intensity of 1000 µmol/m^2^/s. The data for 5 June (day –4) were the result of preliminary measurement for reference (leaf C).

### 4.3. Measurement of Isoprene Emission and Photosynthesis

Isoprene emission and photosynthesis of a leaf of *F. septica* sapling were simultaneously analyzed by an online isoprene analyzer (KFCL-500, Anatec Yanako, Kyoto, Japan) connected with a CI-340 handheld photosynthesis system (CID Bio-Science, Inc., Washington, DC, USA, imported by Ogawa Seiki Co., Tokyo, Japan). Leaves were held in an LC-5 leaf chamber, and measurements of isoprene emission, photosynthesis, leaf temperature, and light intensity were monitored at the same time. Ambient fresh air was pumped into the leaf chamber at a flow rate of 400 mL/min, and the outlet flow from the LC-5 leaf chamber was introduced through a 3-way valve into the isoprene analyzer with a flow rate of 100 mL/min. Isoprene in the outlet flow was reacted with ozone to produce chemiluminescence, and the luminescence intensity was monitored online by a blue-sensitive photomultiplier tube [59].

Saplings were placed in a phytotron (Koito Manufacturing Co. Ltd., Tokyo, Japan), and the leaves were irradiated with LED light (CS Specialized Equipment Co., Sapporo, Japan) with 5 min stepwise variation in light intensity. The lighting program consisted of 13 steps of up and down phases, as shown in Supplementary Figure S1A. At each step, light intensity was held constant for 5 min. Leaf temperature was allowed to vary naturally with light intensity, as shown in Supplementary Figure S1B. The isoprene emission, leaf temperature, and light intensity acquired were averaged for 5 min and used for the optimization of G93 parameters, as described previously [44].

The isoprene analyzer was calibrated with standard isoprene gas (17.52 ppm) purchased from Tokyo Koatsu Co., Tokyo, Japan, as recommended by the supplier: first calibration on high-range mode using 17.52 ppm isoprene standard, followed by manual calibration of low-range mode (200 ppb max). Calibration by this procedure usually gives a minimum detection sensitivity (2 s) of 1.2 ppb for low-range measurements. Isoprene emission was analyzed using the low-range mode, and all measurements were corrected by subtracting the background emission level of isoprene (2–3 ppb) in ambient air. The changes in background level during the measurement were usually less than 1 ppb and remained within the range of minimum detection sensitivity (2 s = 1.2 ppb).

### 4.4. Parameterization of G93

The model G93 estimates isoprene emission (*I*) as

(1) $$I=Is\cdot C_{T}\cdot C_{L}$$

where *I* is the emission rate predicted at temperature *T* (K) and (PPFD) of *L* (µmol/m^2^/s), and *Is* is the basal emission factor (emission factor at standard conditions of *T* = 303 K, *L* = 1000 µmol/m^2^/s) [33]. The two variables *C_T_* and *C_L_* are temperature and light coefficients, respectively, and are defined by

(2) $$C_{T}=\frac{exp\frac{C_{T1}\left(T-T_{s}\right)}{RT_{s}T}}{1+exp\frac{C_{T2}\left(T-T_{M}\right)}{RT_{s}T}}$$

(3) $$C_{L}=\frac{{aC}_{L1}L}{\sqrt{\left(1+a^{2}L^{2}\right)}}$$

where *T* is the measurement temperature (K), *L* is the measured PPFD (µmol/m^2^/s), R is the universal gas constant (=8.314 J K^−1^ mol^−1^), *Ts* is the leaf temperature at standard conditions (303 K), and ***C_T_*_1_** (=95,000 J mol^−1^), ***C_T_*_2_** (=230,000 J mol^−1^), *T_M_* (=314 K), **α** (=0.0027), and *C_L_*_1_ (=1.066) are empirical constants. 

The parameters of ***C_T_*_1_**, ***C_T_*_2_**, and **α** define the amplitude and shape of the temperature and light response curves of the G93 emission model, and the constants listed above are empirical coefficients determined by nonlinear best fit methods using observations from four temperate plant species. Our previous study successfully optimized the G93 parameters for tropical trees using an iterative method named “Ping-Pong” involving mutual step-by-step optimization of the parameters listed above: ***C_T_*_1_**, ***C_T_*_2_**, and **α** [44]. The robustness and accuracy of the “Ping-Pong” method have been further evaluated in our previous studies under different conditions [45,46]. We denote the G93 optimized using Ping-Pong as O-G93 (O-G93) to distinguish it from the default G93 in this paper. The authors herein applied this procedure to parameterize the G93 model and to characterize the temperature and light response of *F. septica* leaves. The convergency criterion of parameters was as described previously [44]. 

### 4.5. Statistical Analysis

The statistical significance between pairs of means was evaluated by the Tukey test using BellCurve for Excel (Version 2312, Social Survey Research Information Co., Ltd., Tokyo, Japan) throughout this study. The results of the post hoc analyses were presented only when the initial ANOVA test yielded statistically significant results. Statistical significance for the correlation coefficients was evaluated by Student’s *t*-test.

## 5. Conclusions

The present study describes the relationship between cumulative temperature and light intensity and the parameters of the G93 algorithm. Cumulative temperature and light intensity of the previous days were found to alter the emission behavior of *F. septica* leaves when illuminated with varying PPFD while allowing leaf temperature to vary naturally with PPFD. It was found that *C_T_*_2_ and α were altered with an increase in cumulative temperature and PPFD, while *C_T_*_1_ did not show any corresponding changes. Although the parameters were optimized at best by the iterative Ping-Pong method, O-G93 displayed limited performance with increasing cumulative temperature at the latter stages after day 8 in this experiment, a deficiency shared by both the default G93 and MEGAN models that also deviated from observed emission flux. The deviation was most pronounced for the PPFD descending phase due to a shift of emission peak, and was solved through separate optimization of the ascending and descending phases especially after the plant experienced hot weather. Measurements using an increased number of tropical plants are necessary in a future study to generalize this observation. This study suggests that the assumption of even efficacy of formula coefficients for both ascending and descending phases of isoprene emission needs revision, particularly when plants have gone through hot weather days. This is particularly important considering the diurnal pattern of isoprene emission that peaks at noon, as simulated by the ascending and descending phases in our design. The present study thus points to the importance of the optimization of formula parameters and the improvement of the model formula because, currently, the bulk of isoprene emission models are empirically based. The observations reported herein have important implications for the improvement of isoprene emission models, particularly under the predicted increase in future global temperatures.

## Figures and Tables

**Table 1:** Correlation between average temperature of past days and optimized G-39 parameters.

Past Days	*C_T_*_1_	*C_T_*_2_	α
1	0.01	0.32	**0.65**
2	0.02	**0.44**	**0.40**
3	0.04	**0.61**	0.40
4	0.05	**0.64**	**0.52**
5	0.07	**0.65**	**0.73**
6	0.10	**0.52**	**0.87**
7	0.13	**0.44**	**0.90**
8	0.16	**0.46**	**0.78**
9	0.20	**0.41**	**0.42**
10	0.18	0.33	0.05

Correlation coefficients in bold denotes statistical significance at *p* < 0.05.

**Table 2:** Correlation between average PPFD of past days and optimized G-93 parameters.

Past Days	*C_T_*_1_	*C_T_*_2_	α
1	−0.21	0.24	0.09
2	0.07	0.30	0.06
3	0.09	0.28	−0.13
4	−0.03	0.35	−0.19
5	−0.13	**0.42**	−0.11
6	−0.09	0.31	0.14
7	0.11	0.30	0.07
8	0.21	−0.01	**−0.14**
9	0.14	−0.27	**−0.57**
10	0.05	−0.09	**−0.65**

Correlation coefficients in bold denotes statistical significance at *p* < 0.05.

**Table 3:** Correlation between average temperature of past days and MEP metabolite, *IspS* gene expression, and *IspS* protein level.

Past Days	DXP	MEP	MEcDP	HMBDP	DMADP	*IspS*	IspS Protein
1	**0.39**	**0.49**	**0.47**	0.36	0.36	**0.57**	0.27
2	0.36	**0.40**	**0.44**	0.32	0.37	**0.52**	0.28
3	0.18	0.32	0.28	0.16	**0.37**	**0.45**	0.32
4	−0.05	0.23	0.13	0.05	0.33	0.34	0.35
5	−0.27	0.18	0.01	−0.02	0.33	0.24	0.37
6	−0.33	0.12	0.01	0.03	0.27	0.16	0.33
7	−0.29	0.07	0.05	0.07	0.26	0.17	0.32
8	−0.24	0.00	0.07	0.05	0.22	0.18	0.30
9	−0.15	−0.14	−0.02	−0.09	0.10	0.14	0.24
10	−0.14	−0.26	−0.19	−0.26	−0.05	0.01	0.13

Correlation coefficients in bold denotes statistical significance at *p* < 0.05.

**Table 4:** Correlation between average PPFD of past days and MEP metabolite, *IspS* gene expression, and *IspS* protein level.

Past Days	DXP	MEP	MEcDP	HMBDP	DMADP	*IspS*	IspS Protein
1	0.38	0.46	0.51	**0.42**	0.30	**0.45**	0.14
2	0.35	0.29	0.37	0.25	0.27	**0.40**	0.19
3	0.21	0.08	0.12	−0.01	0.15	0.24	0.14
4	0.01	0.00	−0.10	−0.17	0.02	0.04	0.07
5	−0.27	−0.07	−0.29	−0.30	−0.09	−0.16	−0.01
6	**−0.40**	−0.35	−0.32	−0.24	−0.30	**−0.47**	−0.17
7	**−0.39**	**−0.53**	**−0.38**	−0.29	−0.27	**−0.51**	−0.16
8	−0.37	**−0.53**	**−0.43**	−0.37	−0.37	**−0.54**	−0.23
9	−0.11	**−0.55**	−0.30	−0.27	**−0.42**	**−0.47**	−0.28
10	−0.10	**−0.49**	−0.33	−0.32	−0.35	**−0.43**	−0.28

Correlation coefficients in bold denotes statistical significance at *p* < 0.05.

**Table 5:** Comparison of G-93 parameters between ascending and descending phases of isoprene emission phases.

Parameter	Whole (Steps 1–13)	Ascending (Steps 1–7)	Descending (Steps 8–13)
*C_T_*_1_	196,750 ± 4553 ^a^	189,250 ± 8199 ^a^	47,500 ± 5545 ^b^
*C_T_*_2_	237,000 ± 2646 ^a^	249,750 ± 4250 ^a^	280,250 ± 5344 ^b^
α	0.0851 ± 0.0002 ^a^	0.0034 ± 0.0004 ^b^	0.0039 ± 0.0002 ^b^
BER	10.6 ± 0.4 ^a^	9.7 ± 0.7 ^a^	28.6 ± 2.1 ^b^

BER: Basal emission rate. Data not sharing the same superscript letter are significantly different at *p* < 0.05.

## Data Availability

Data are contained within the article and Supplementary Materials.

## Supplementary Materials

The following supporting information can be downloaded at: https://www.mdpi.com/article/10.3390/plants13020243/s1.
