# Supplementary material for: Relationship between Cumulative Temperature and Light Intensity and G93 Parameters of Isoprene Emission for the Tropical Tree Ficus septica
Source: Plants (Basel). 2024 Jan 15;13(2):243. doi: 10.3390/plants13020243 (PMC10820733; doi:10.3390/plants13020243)
Supplement: Supplementary file 1 [file plants-13-00243-s001.zip › plants-2751186-supplementary.pdf]

Supplementary Materials

Table S1. Comparison of G93 parameters between ascending and descending phases of isoprene emission on day 3.

Parameter	Whole (Steps 1–13)	Ascending (Steps 1–7)	Descending (Steps 8–13)
CT1	192,500 ± 17,265 [a]	138,000 ± 10,432 [b]	106,000 ± 2708 [b]
CT2	207,000 ± 1080 [a]	273,000 ± 55,368 [a]	243,500 ± 8067 [a]
α	0.0059 ± 0.0007 [a]	0.0022 ± 0.0005 [b]	0.0030 ± 0.0002 [b]
BER	14.3 ± 1.3 [a]	18.3 ± 2.0 [a]	22.8 ± 3.0 [a]

BER: basal emission rate. Data not sharing the same superscript letter are significantly different at $p < 0.05$.

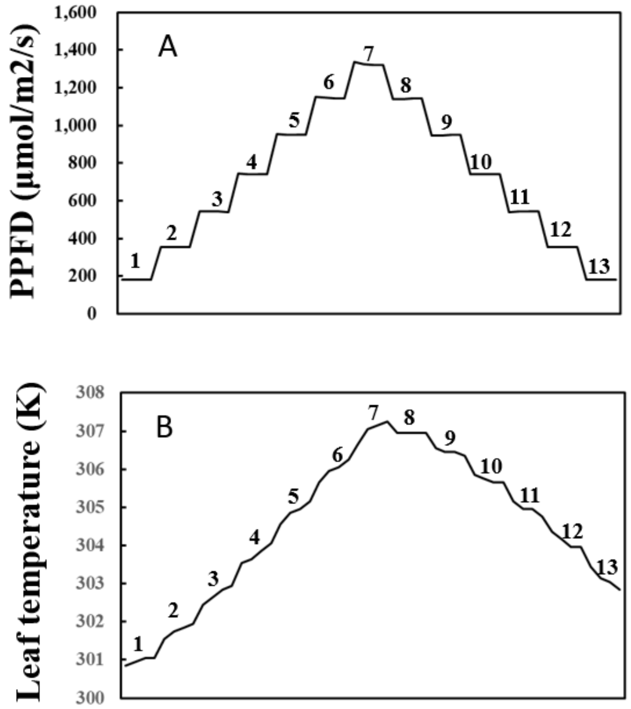

Figure S1. Light regime of "Ping-Pong" method (**A**) and typical change in leaf temperature (**B**). Light intensity was changed from 180 to 1,300 µmol/m^2/s with 5 min interval and 1 round of up and down phase.

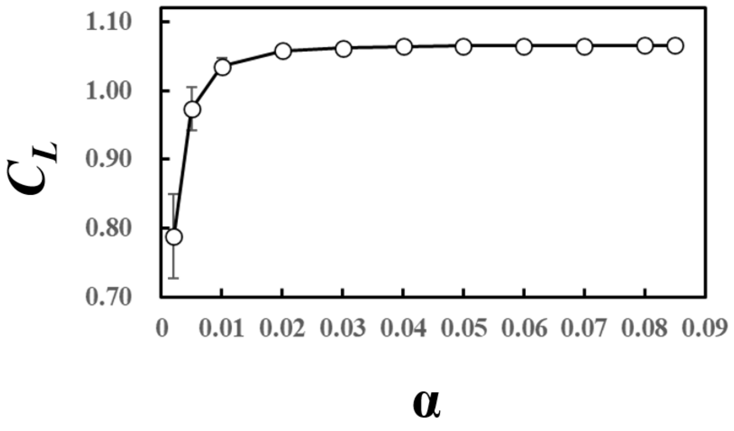

Figure S2. Relationship between α and CL. Data are mean± SE of lighting regime used for "Ping-Pong" method as indicated in Supplementary Figure S1.

Supplementary Document S1

γP and γT of MEGAN 2.1 for isoprene emission (Guenther et al., 2012)

$\gamma P = C_P[(\alpha \times \mathrm{PPFD})/((1+\alpha^2 \times \mathrm{PPFD}^2)^{0.5})]$
where
$\alpha = 0.004 - 0.0005\ln(P_{240})$

$C_P = 0.0468 \times \exp(0.005 \times [P_{24} - P_S]) \times [P_{240}]^{0.6}$
where
Ps (standard conditions for PPFD for sun leaves) is 200 μmol $m^{-2}s^{-1}$
P_{24} and P_{240} is the averaged PPFD of the past 24h and 240h respectively

$\gamma T = E_{opt} \times [C_{T2} \times \exp(C_{T1} \times x)/(C_{T2} - C_{T1} \times (1 - \exp(C_{T2} \times x)))]$
where
$x = [(1/T_{opt}) - (1/T)]/0.0083$
T is leaf temperature (K), C_{T1}=95, C_{T2}=230

$T_{opt} = 313 + (0.6 \times (T_{240} - T_S))$
$E_{opt} = 2 \times \exp(0.05 \times (T_{24} - T_S)) \times \exp(0.05 \times (T_{240} - T_S))$
Where T_S (standard conditions for average leaf temperature) is 297K
T_{24} and T_{240} is the average temperature of the past 24h and 240h respectively
